# Supplementary material for: The Transcriptomic Profiles of ESR1 and MMP3 Stratify the Risk of Biochemical Recurrence in Primary Prostate Cancer beyond Clinical Features
Source: Int J Mol Sci. 2023 May 7;24(9):8399. doi: 10.3390/ijms24098399 (PMC10179071; doi:10.3390/ijms24098399)
Supplement: Supplementary file 1 [file ijms-24-08399-s001.zip › ijms-2365172-supplementary.pdf]

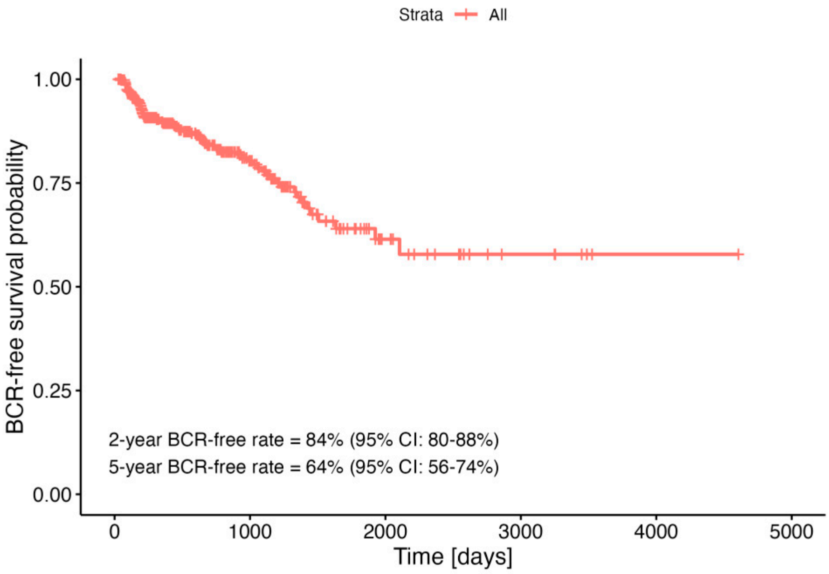

Supplementary Figure S1. BCR-free survival of the TCGA PRAD cohort.

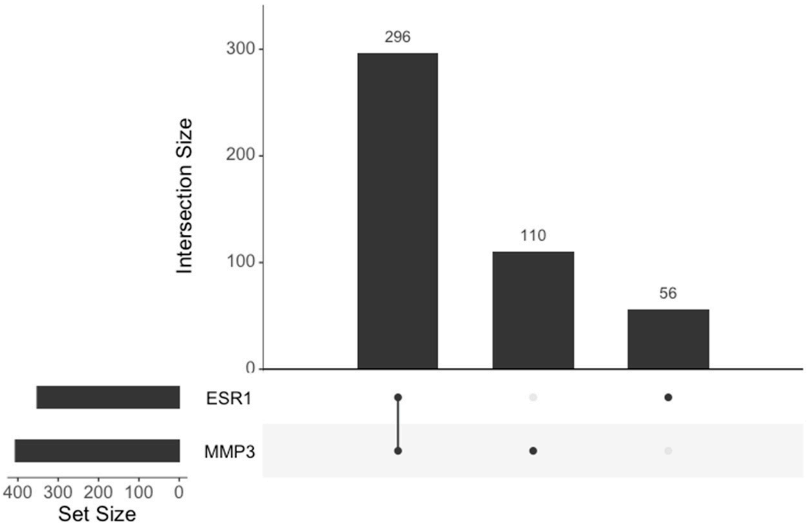

Supplementary Figure S2. The UpSet plot revealed a subgroup of 296 PRAD patients sharing unfavorable molecu-lar signature of ESR1 and MMP3 altering the BCR-free survival (hereinafter called the “strata”).

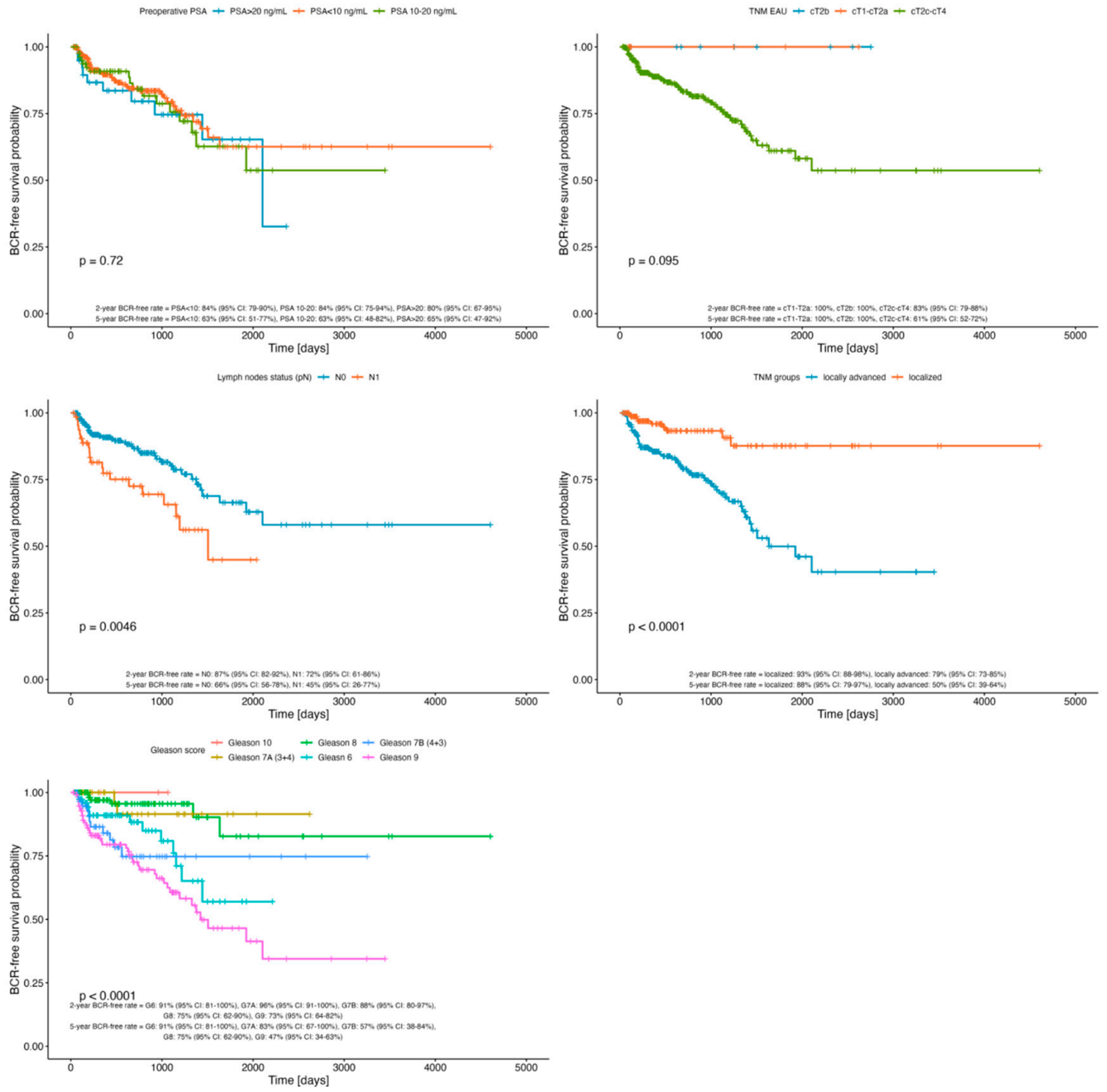

Supplementary Figure S3. Kaplan-Meier curves showing significant differences in BCR-free rates regarding lymph nodes status (p = 0.0046), TNM groups (p < 0.0001) as well as Gleason score (p < 0.0001), in contrast to groups of preoperative PSA and TNM catgories by EAU, which did not stratify BCR-free survival of PRAD patients in a significant manner (p = 0.72, p = 0.095, respectively).

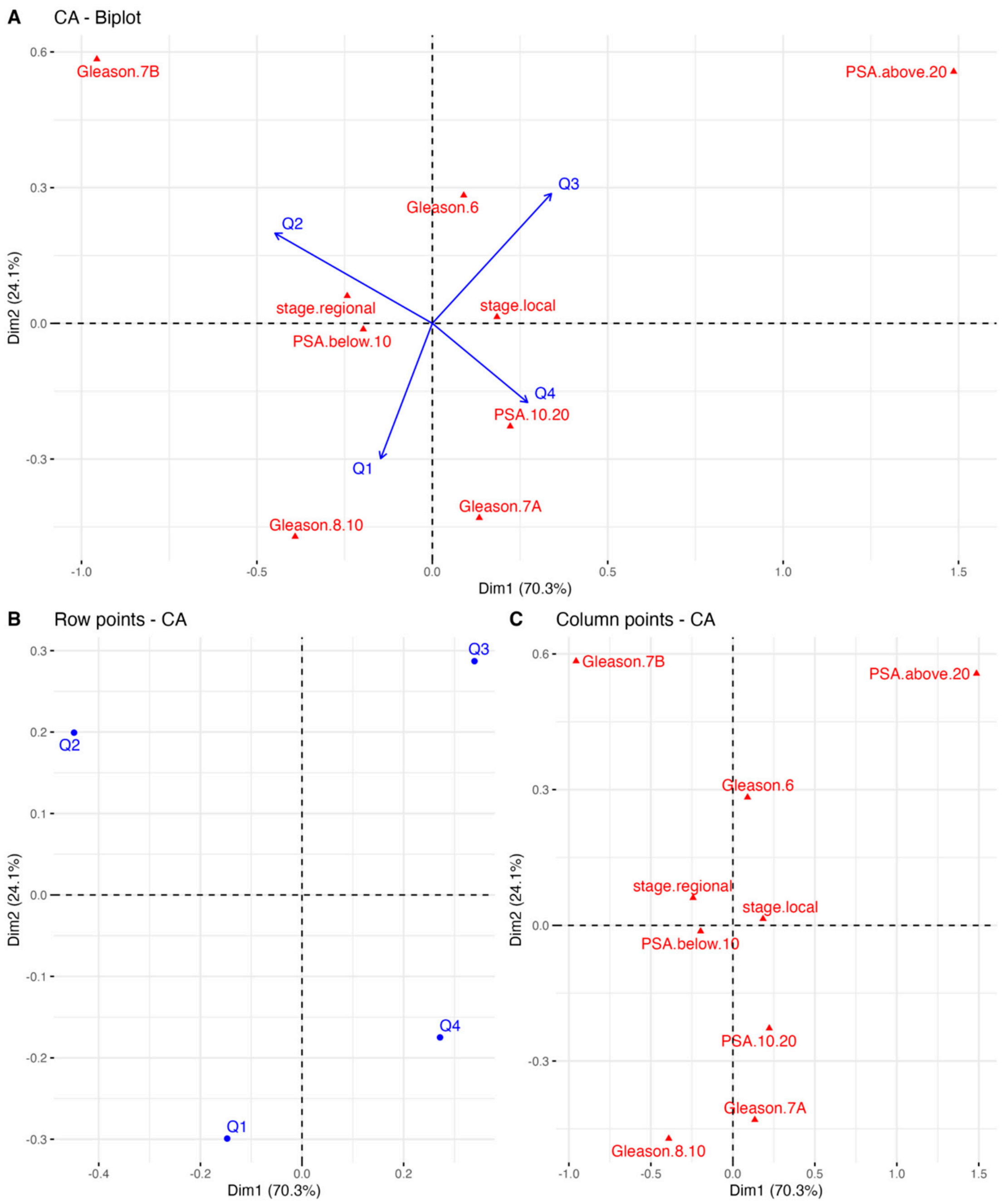

Supplementary Figure S4. The CA plot for cross-validation data of the **A**) representation of both, expression quantiles of *ESR1* and *MMP3* and clinical typologies, **B**) only row variables, i.e. quantiles, and **C**) the distribution of the column points only, i.e. clinical variables, partitioned along the two first dimensions of total variability reaching 94.4%.

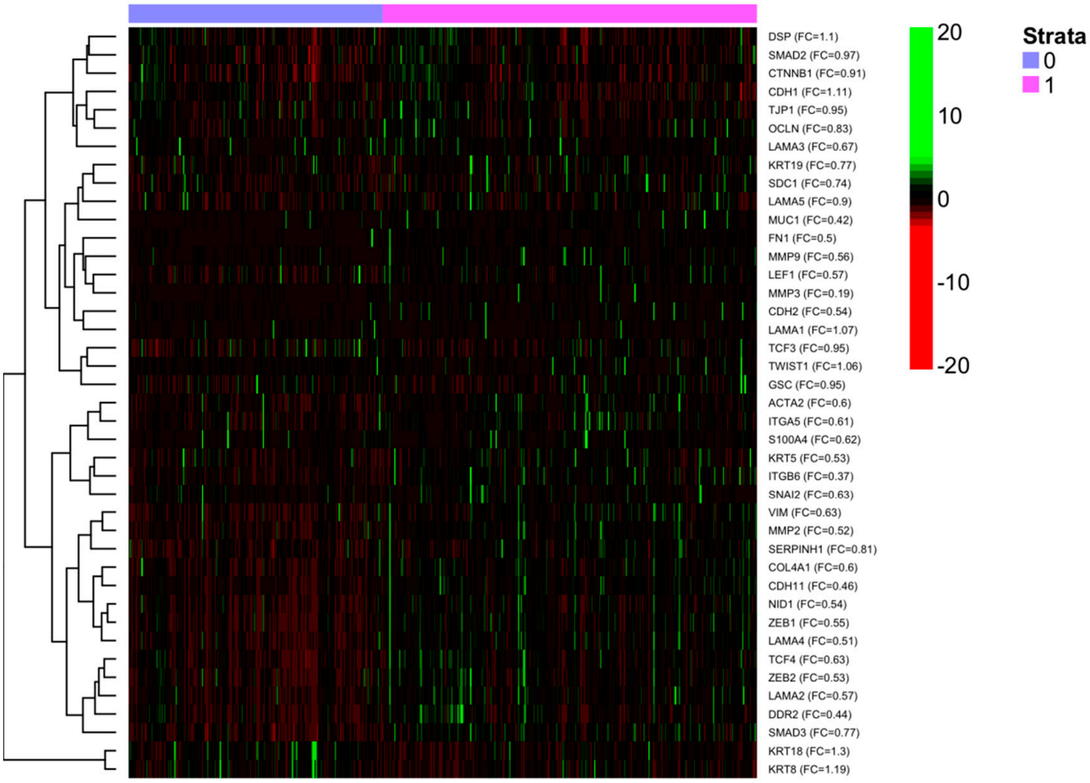

Supplementary Figure S5. The transcriptomic profiling of the 41 major EMT markers among PRAD patients grouped by the identified strata (unfavorable expression profiles of *ESR1* and *MMP3*). Additionally, for each gene fold change (FC) of expression between strata-negative and strata-positive subgroups has been calculated.
